# Unexpected Patterns of Admixture in German Populations of *Aedes japonicus japonicus* (Diptera: Culicidae) Underscore the Importance of Human Intervention

**DOI:** 10.1371/journal.pone.0099093

**Published:** 2014-07-03

**Authors:** Dorothee E. Zielke, Doreen Werner, Francis Schaffner, Helge Kampen, Dina M. Fonseca

**Affiliations:** 1 Leibniz-Centre for Agricultural Landscape Research, Müncheberg, Germany; 2 National Centre for Vector Entomology, Institute of Parasitology, University of Zurich, Zurich, Switzerland; 3 Avia-GIS, Zoersel, Belgium; 4 Friedrich-Loeffler-Institut, Federal Research Institute for Animal Health, Greifswald – Insel Riems, Germany; 5 Center for Vector Biology and Department of Entomology, Rutgers University, New Brunswick, New Jersey, United States of America; Universidade Federal do Rio de Janeiro, Brazil

## Abstract

The mosquito *Aedes japonicus japonicus*, originally restricted to temperate East Asia, is now widespread in North America and more recently has become established in Europe. To ascertain the putative number of separate introductions to Europe and examine patterns of expansion we analyzed the genetic makeup of *Ae. j. japonicus* populations from five cemeteries in North Rhine-Westphalia and Rhineland-Palatinate, two western German federal states, as well as of specimens from populations in Belgium, Switzerland, and Austria/Slovenia. To do so, we genotyped individual specimens at seven pre-existing polymorphic microsatellite loci and sequenced part of the *nad*4 mitochondrial locus. We found evidence of two different genotypic signatures associated with different *nad*4 mitochondrial haplotypes, indicating at least two genetically differentiated populations of *Ae. j. japonicus* in Europe (i.e. two distinct genotypes). Belgian, Swiss, and Austrian/Slovenian populations all share the same genotypic signature although they have become differentiated since isolation. Contrary to expectations, the German *Ae. j. japonicus* are not closely related to those in Belgium which are geographically nearest but are also highly inbred. German populations have a unique genotype but also evidence of mixing between the two genotypes. Also unexpectedly, the populations closest to the center of the German infestation had the highest levels of admixture indicating that separate introductions did not expand and merge but instead their expansion was driven by punctuated human-mediated transport. Critically, the resulting admixed populations have higher genetic diversity and appear invasive as indicated by their increased abundance and recent spread across western Germany.

## Introduction

Biological invasions of potential disease vectors such as mosquitoes (Diptera: Culicidae) inflict more direct threat to human health than those of many other species because they can radically alter the frequency of local or exotic pathogen transmission to humans and wildlife [1]. Notably, invasive mosquitoes are the leading drivers of historic epidemics of yellow fever and of contemporary epidemics of West Nile fever, dengue, and chikungunya [2], [3]. The contemporaneous worldwide movement of humans and goods has increased the rate of introductions and establishment of exotic mosquitoes [4], especially those with desiccation resistant eggs, such as many in the genus *Aedes* [5].

A very recent expansion is that of *Aedes japonicus japonicus* Theobald, 1901, also known as the Asian bush mosquito. Although intercepted a few times in the early nineties in New Zealand’s ports [6], the first established populations outside the original distribution range were detected in two eastern states of the United States of America (US) in 1998 [7] from where they have quickly spread to over 30 eastern US states [8]–[10]. Today, the species is present in both US coasts and Canada [11], [12] and although this subspecies of *Ae. japonicus*, one of four, is originally restricted to climates with cold snowy winters in Japan and Korea [13], [14] surprisingly it became established in the Hawaiian Islands in 2003 [15].

Of note, the current generic name of this species is controversial as it is often called *Ochlerotatus japonicus* and, more recently, *Hulecoeteomyia japonica* [16]. We are using *Aedes japonicus* following the guidelines of Edman and colleagues [17].

In Europe, the first detection of *Ae. j. japonicus* occurred in 2000 when larvae in water in tires at a used tire import platform in Normandy, France, were eliminated before adult emergence [18]. However, in 2002, the species was detected at one location in Belgium, again on a used tire import platform, in Namur province. It was still there in 2003 and 2004 as well as in 2008 during a national mosquito monitoring campaign when it was also documented on a second used tire platform 2 km away [19]. Because *Ae. j. japonicus* has never been caught outside these two locations, the populations were considered to be not expanding. Nonetheless, in 2012 a control campaign was started aiming at eliminating the species, although to our knowledge it is unclear if it succeeded. In 2008, larvae of *Ae. j. japonicus* were also found in northern Switzerland and in several locations near the border on the German side [20]. A monitoring program carried out in 2009 and 2010 in the affected German federal state of Baden-Württemberg showed the species already occurring over a large area along the border with Switzerland [21]. Another study in 2010 demonstrated a punctual appearance near the city of Stuttgart, approximately 80 km north of the previously known northern distribution border of the species [22]. In the same year, *Ae. j. japonicus* was also found distributed across a 50 km area around the Austrian-Slovenian border [23]. The origins of all these discontinuous populations have remained a mystery so far, but in Germany further *Ae. j. japonicus* infestations have been recognized and the species is now prevalent in the northern part of the federal state of Rhineland-Palatinate and the southern part of the state of North Rhine-Westphalia up to the city of Cologne [24].

Because *Ae. j. japonicus* is a potential vector of several human encephalitis viruses as well as of dengue, chikungunya and Rift Valley fever viruses [20], [25], [26], it is important to understand the routes of introduction and expansion of the species in Europe. Compared to the Asian tiger mosquito *Aedes albopictus*, *Ae. j. japonicus* is adapted to colder climate, which explains why the latter occurs in northern Europe while the former remains restricted to southern Europe and so far is only found occasionally in Southwest Germany [27], [28]. Although the introduction of a new species to a region is always a risk, it also provides an excellent opportunity to observe important drivers of population growth and dispersion that are difficult to detect in existing populations [9]. Knowledge about the number of introductions and modes of spreading after introduction of an invasive species is important to make predictions on future movements and to decide on appropriate control measures [29].

Highly polymorphic DNA regions such as those associated with simple sequence repeats (SSR, also called microsatellites) are powerful tools for studying populations of introduced species both by revealing putative origins as well as changes in allelic frequencies through space and time [30]. Shifts in genetic makeup associated with introductions can be measured by comparison of populations across the exotic range or with those in the original range. These genetic changes can be substantial and have unexpected behavioral or physiological consequences [31]–[33]. Additionally, analyzing the genetic makeup of a newcomer may help us predict the invader’s ability to become established successfully, a trait that can be greatly influenced by genetic variability [34].

The objective of the present study was to examine the patterns of expansion of *Ae. j. japonicus* in North Rhine-Westphalia and Rhineland-Palatinate in western Germany. Specifically, we aimed to answer the following questions: Did the West German mosquitoes originate from nearby existing populations in Belgium, or from populations farther away in Switzerland or Austria/Slovenia, or did other introduction events take place? If multiple introductions occurred are they remaining separate or are they mixing? How are German *Ae. j. japonicus* populations expanding?

## Materials and Methods

### Mosquito collections

Because adult male or female *Ae. j. japonicus* are not efficiently attracted to standard traps used in mosquito surveillance [35], to the best of our knowledge the most reliable way to collect this species is as larvae from small water holding containers, which was the strategy used across all locations included in this study. Since cemeteries generally have many small water containers such as flowerpot pans and vases, they are ideal habitats for mosquitoes that utilize containers for immature development such as many *Aedes* and *Culex* species [36]. Cemeteries are also hotspots for mosquito production because adults find shelter in those park-like sites, where a profusion of plants provide protective moist habitats as well as sugary nutrition to both males and females [36].

**Figure 1:**
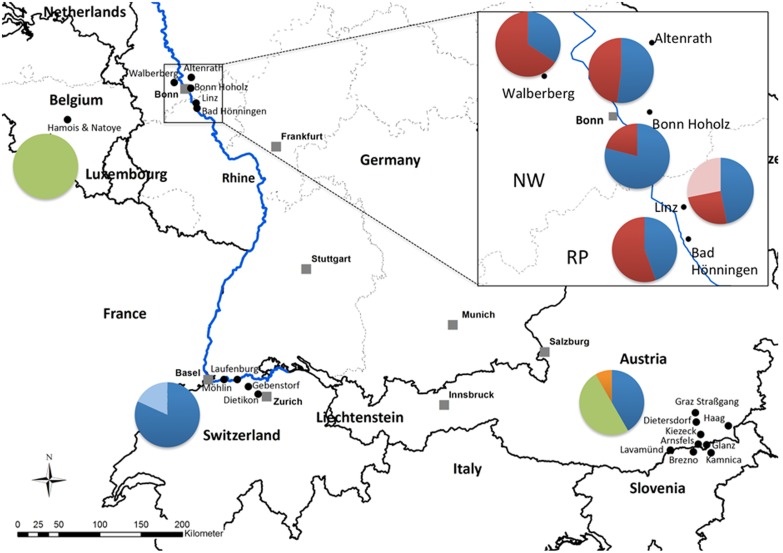
Sampling spots in North Rhine-Westphalia and Rhineland-Palatinate in Germany (box) as well as in Belgium, Switzerland, and Austria/Slovenia. Pie charts show the relative frequency of the six *nad*4 mitochondrial DNA haplotypes. NW = North Rhine-Westphalia, RP = Rhineland-Palatinate. Haplotypes: blue = H1, red = H5, pink = H6, green = H9, orange = H10, light blue = H33. The numbers of specimens sequenced at the *nad*4 locus in each population are listed in Table 1.

In August 2012, after several specimens of *Ae. j. japonicus* had been sent to us at ZALF in the framework of the online project “Mückenatlas” (http://www.mueckenatlas.de), we started a monitoring program focusing on cemeteries in the states of North Rhine-Westphalia and Rhineland-Palatinate (western Germany). In every cemetery sampled, *Ae. j. japonicus* larvae were collected from at least seven water containers to avoid over-sampling across siblings which would potentially bias the genetic analyses. Larvae were similarly sampled in Switzerland, while in Belgium they were collected from several used tires. Specimens from Austria/Slovenia were obtained from various containers scattered on the side of roads. The mosquitoes were processed as described in Kampen and colleagues [24]. All collection locations are shown in Figure 1 with more details in Table 1.

**Table 1:** Sampling spots in Belgium, Germany, Switzerland, Austria and Slovenia, listed in order of decreasing latitude.

Federal state or province/canton or statistical region	Location	Latitude	Longitude	Date	N_S_	N_m_
Namur, **Belgium**	Natoye, Hamois	50.3389 N	05.0447 E	08/14/2008	6	6
				08/31/2010	12	12
North Rhine-Westphalia, **Germany**	Altenrath	50.8597 N	07.1959 E	08/29/2012	25	35
	Walberberg	50.7933 N	06.9094 E	08/23/2012	32	40
	Bonn-Hoholz	50.7365 N	07.1979 E	08/23/2012	38	40
Rhineland-Palatinate, **Germany**	Linz	50.5748 N	07.2962 E	08/23/2012	32	41
	Bad Hönningen	50.5197 N	07.3104 E	08/24/2012	27	41
Aargau, **Switzerland**	Möhlin	47.5696 N	07.8256 E	06/29/2012	7	7
	Laufenburg	47.5561 N	08.0611 E	09/04/2008	3	3
	Gebenstorf	47.4842 N	08.2386 E	08/28/2008	6	6
Zurich, **Switzerland**	Dietikon	47.3979 N	08.4060 E	09/02/2008	2	6
Südweststeiermark, **Austria**	Graz Straβgang	47.0219 N	15.3984 E	09/23/2011	2	2
	Dietersdorf	46.9197 N	15.4021 E	09/10/2011	2	2
	Kitzeck	46.7814 N	15.4541 E	09/10/2011	4	4
	Arnfels	46.6762 N	15.4037 E	09/24/2011	5	5
	Glanz	46.6620 N	15.5355 E	09/10/2011	2	2
Steiermark, **Austria**	Haag	46.8477 N	15.9046 E	08/30/2011	5	5
Kärnten, **Austria**	Lavamünd	46.6358 N	14.9539 E	09/24/2011	6	6
Koroška, **Slovenia**	Brezno	46.5961 N	15.3169 E	09/24/2011	5	4
Drava, **Slovenia**	Kamnica	46.5718 N	15.5965 E	09/24/2011	6	6

**Figure 2:**
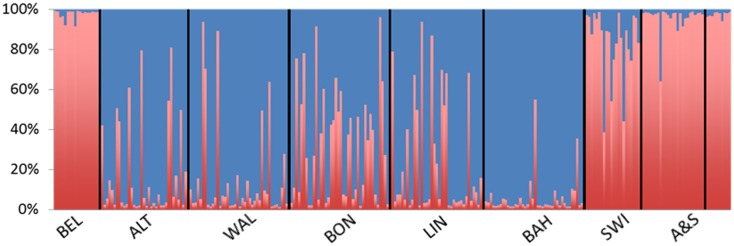
Results of a Bayesian cluster analysis of multilocus microsatellite genotypes. Each individual included in the analysis is represented by a thin vertical line, partitioned into colored segments that represent the individual’s probability of belonging to one of the two most likely genetic clusters. The origin of each specimen was not used in the analysis. Red = genotype 1, green = genotype 2, BEL = Belgium; ALT = Altenrath; WAL = Walberberg; BON = Bonn-Hoholz; LIN = Linz; BAH = Bad Hönningen; SWI = Switzerland; A&S = Austria/Slovenia. One specimen from Bad Hönningen has a genotype indicating it is likely the result of a cross between genotype 1 and genotype 2 (an F1).

A total of 227 individuals were sequenced at *nad*4 (N_S_) and 273 were genotyped at 7 microsatellite loci (N_m_). “Date” refers to the day of collection from the field. The same order of specimens was used in the Bayesian multilocus genotype analysis (Fig. 2).

### Mosquito identification and DNA extraction

Larvae from German sites were brought to the laboratory and reared to adults, then killed by exposing them to −20°C for a few minutes, and identified morphologically to species using the key developed by Schaffner and colleagues [37]. Identified adults were stored frozen at −20°C. To confirm the morphological identification, DNA from at least one mosquito from every location was extracted and the CO1 region was sequenced following the protocols of Kampen and colleagues [38] and compared to *Aedes j. japonicus* CO1 sequences in GenBank. Specimens from Belgium, Switzerland and Austria/Slovenia were killed as larvae, stored in alcohol (70%) and identified using the dichotomous key from Schaffner [39] and the multiple-entry key from Schaffner and colleagues [37].

We examined individuals from five cemeteries in western Germany, one tire-recycling platform in Belgium, five locations in Switzerland and eight locations in Austria/Slovenia (Fig. 1, Table 1). We extracted DNA from individual whole adult or larval mosquitoes using either a QIAamp DNA Mini Kit or a DNeasy Blood & Tissue Kit (both from Qiagen) and re-suspended the DNA in 80 µl of buffer EB (Qiagen).

### *Nad4* sequencing

We sequenced a 424-bp fragment in the sodium dehydrogenase subunit 4 (*nad*4) region of the mosquito mitochondrial DNA (between positions 8398 and 8821 in the *Anopheles gambiae* complete mitochondrial genome sequence, GenBank accession #L20934, [8]) that has shown to be variable and informative for population level analyses [8]. We used primers ND4F 5′-CGTAGGAGGAGCAGCTATATT-3′ and ND4R1X 5′-TGATTGCCTAAGGCTCATGT-3′ [40]. Amplifications were performed in a Bio-Rad C1000 thermal cycler (Bio-Rad Laboratories). Amplifications were preceded by a five minute denaturation at 96°C and consisted of 35 cycles of 40 s at 94°C, 40 s at 56°C and 40 s at 72°C, followed by a final extension step of five minutes at 72°C. PCR products were gel-electrophoresed, excised from the gels and recovered with a QIAamp Gel Extraction Kit (Qiagen). They were then cycle-sequenced in both directions with a BigDye Terminator v1.1 Cycle Sequencing Kit (Applied Biosystems/Life Technologies) using one of the amplification primers, then purified with SigmaSpin Sequencing Reaction Clean-Up Columns (Sigma-Aldrich) and run on a 3130 Genetic Analyzer (Applied Biosystems/Life Technologies).

### Microsatellite analysis

We amplified seven microsatellite loci currently available for *Ae. j. japonicus* [41] using a re-designed OJ5F primer [9]. PCR amplifications were performed in Veriti 96-Well Thermal Cyclers (Applied Biosystems/Life Technologies). The PCR profile was comprised of 30 cycles of 30 s at 94°C, 30 s at 56°C and 30 s at 72°C, preceded by a 5 minute denaturation at 96°C and followed by a 10 minute extension at 72°C. The PCR products were run in an ABI3130XL Genetic Analyzer (Life Technologies) and binned and sized with GeneMapper 3.7 (Applied Biosystems/Life Technologies) using bins optimized on worldwide populations of *Ae. j. japonicus* (Fonseca, unpublished data).

### Statistical analysis

We examined departures from Hardy-Weinberg and obtained Shannon’s information index (*I*), mean number of alleles (N_a_), observed heterozygosity (*H*o), and unbiased expected heterozygosity (u*H*_e_) for each population in GenAlEx 6.5 [42]. Shannon’s information index is a diversity measure that takes into consideration the frequency of each allele in addition to the total number of alleles [43], [44]. We also assigned individuals to putative populations based on the expected frequencies of their genotypes in those populations using a “leave one out” option in GenAlEx 6.5 [45], [46].

To uncover genetic discontinuities among specimens, we examined the relationships between individual multi-locus signatures using a Bayesian approach in STRUCTURE [47] and determined the optimal number of clusters (*K*) using the method of Evanno et al. [48] implemented in STRUCTURE HARVESTER [49]. We also performed a factorial correspondence analysis in Genetix 4.05 [50]. We used the GenAlEx software to calculate population based *F*_ST_ values and Nei’s index of divergence (both biased and unbiased), that formed the distance matrices analyzed with a principal coordinate analysis and were used in Mantel tests to examine the relationship between genetic and geographic distances. Pairwise *F*_ST_ values were also calculated with FSTAT 1.2 in order to check for significance using Fisher exact tests [51].

The distribution of *nad4* haplotypes matches to some extent the distribution of the two genotypes. Specifically, haplotype H5 occurs exclusively and is highly abundant in populations with a predominant genotype 2 signature (Fig. 1).

## Results

We genotyped a total of 273 specimens from four European countries and obtained 227 *nad*4 sequences (Table 1). We identified six *nad*4 haplotypes in our samples: H1, H5, H6, H9, H10, H33 (Fig. 1) that can be reconstructed from GenBank accession no. AF305879 and [8], and accession no. KJ958405 (Fonseca, unpublished data). Strikingly, H5 and H6 occurred only in German populations and H6 was restricted to Linz. In contrast, haplotype H1 was found broadly across all populations except in Belgium where H9 was the only haplotype detected. Haplotype H9 also occurred in Austria/Slovenia together with H10, and H33 occurred in Swiss specimens only (Fig. 1).

The microsatellite multilocus genotype signatures of the specimens fell into two groups (Fig. 2). Specimens from Belgium and Austria/Slovenia had almost exclusively a signature from one group, henceforth named genotype 1, since they were the first found in Europe. Specimens from Bad Hönningen, the southernmost location examined in Germany (Table 1) had almost exclusively a signature from a second group, henceforth named genotype 2. Specimens from the remaining four German populations showed predominantly a signature from genotype 2 but with clear evidence of admixture with genotype 1. Conversely, specimens from Switzerland showed predominantly signatures from genotype 1 but with evidence of some admixture with genotype 2. Microsatellite raw data generated within this study can be obtained from DF.

To increase statistical power [52] in non-individual based microsatellite analyses we combined the specimens from Austria and Slovenia and those from Switzerland into single populations although they were collected from a variety of locations ranging from 10 to 50 km from each other (Fig. 1). We checked for departures from Hardy-Weinberg (H&W) frequencies and found significant departures at OJ10 in Austria/Slovenia and at OJ10 and OJ85 in the Swiss population. However, we found similar levels of incidence of significant departures from H&W frequencies at some loci in the German populations and in Belgium that were derived from specimens collected in the same cemetery or tire platform indicating a lack of biological significance to this small number of random departures from H&W. Departures were both due to significantly lower than expected and higher than expected heterozygosity values, and close inspection did not indicate a significant effect of null alleles [53].

**Figure 3:**
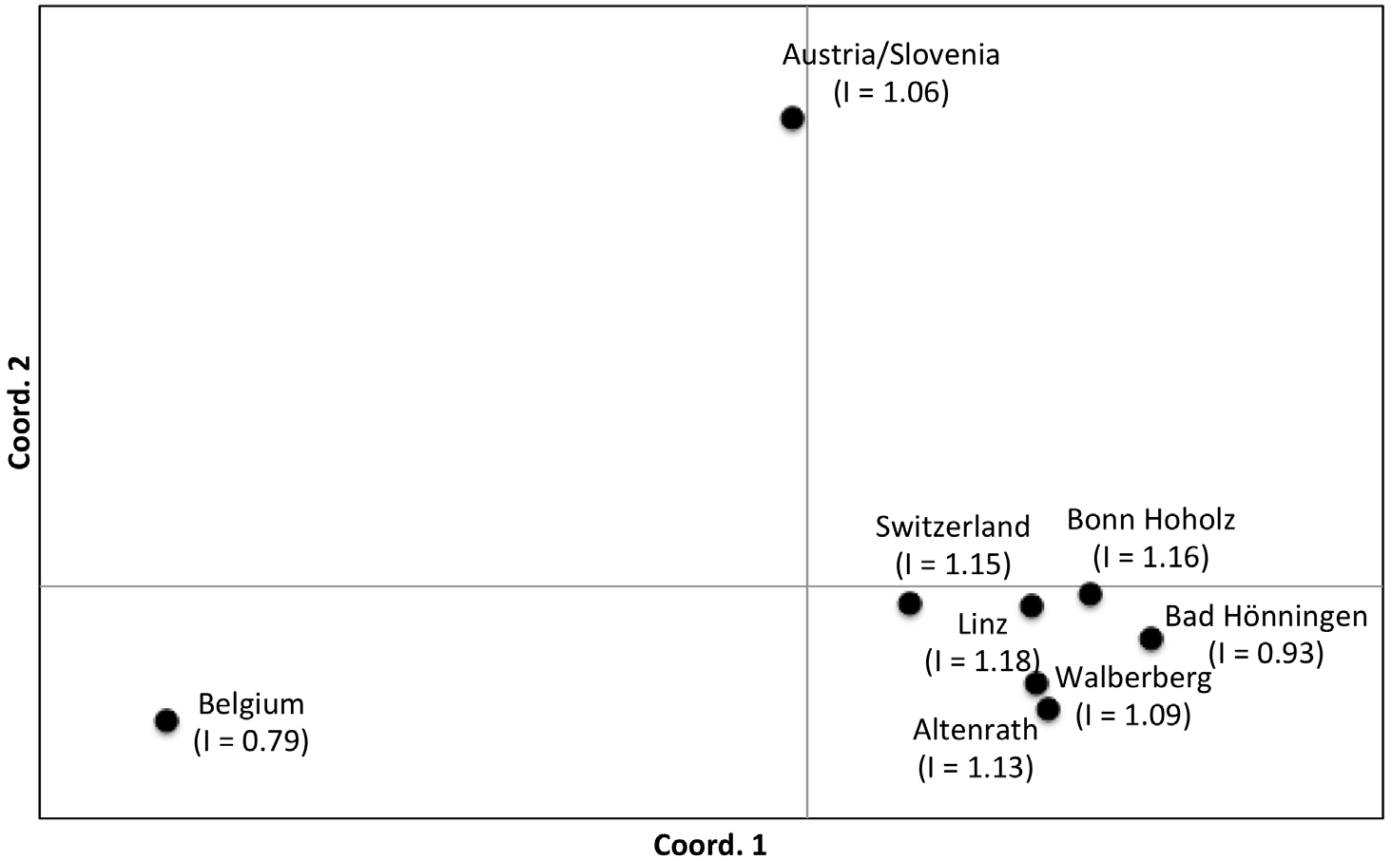
Principal coordinate analysis plot of pairwise *F*_ST_ genetic distances for the five German populations and samples from Belgium, Switzerland, and Austria/Slovenia. For sample sizes please refer to Table 1. Values in parentheses are Shannon diversity indices (I). Coordinates 1 and 2 account for 86.6% of the variation.

Two genetic groups were also identified by assignment tests based on allelic frequency, likelihood, and genetic distance although there was evidence of strong differentiation among locations with a genetic signature from genotype 1 as evidenced by the lack of assignment of specimens to locations outside their own (Table 2). This differentiation among Belgian, Austrian/Slovenian and Swiss populations is also evident from the results of the principal coordinate analyses (Fig. 3) based on pairwise *F*_ST_ values. As would be expected from their proximity, most German populations are closely related although overall the relationship between genetic distance and geographic distance is not significant (Mantel tests, *P*>0.05). Instead, the two populations, Linz and Bonn-Hoholz, closer to the geographic “middle” of the five German sites, have more signs of admixture with genotype 1 and are therefore more similar to Swiss specimens than to the Bad Hönningen population, which is further south and therefore geographically closer to Switzerland (Figs. 2 and 3). Of note, equivalent results were obtained with other measures of pair-wise genetic distance/similarity such as Nei’s indices of divergence (data not shown) as well as from the factorial correspondence analysis based on individual genotypes (Fig. S1).

**Table 2:** Results of the assignment tests.

Population	BEL	WAL	ALT	BON	LIN	BAH	SWI	A&S	SELF%
**Belgium (BEL)**	18								100
**Walberberg (WAL)**		26	5	2	6	1			65
**Altenrath (ALT)**		4	12	6	7	5	1		34
**Bonn Hoholz (BON)**		7	8	6	8	8	3		15
**Linz (LIN)**		5	3	8	9	8	4		24
**Bad Hönningen (BAH)**				2	2	36			90
**Switzerland (SWI)**		3				1	16	2	73
**Austria/Slovenia (A&S)**	1	1				1	3	29	83

“SELF%” is the rate of self-assignment to the population of origin. The actual numbers of specimens self-assigned are highlighted with small boxes on the diagonal. Populations within the center box are from western Germany.

## Discussion

The expanding populations in Germany show a signature of admixture reminiscent of the mixing across Pennsylvania of two separate introductions to the US [9]. Unlike in Pennsylvania where the mixing appeared to occur as the introductions abutted, in western Germany it appears the admixture between the two introductions occurred from the center of the sampled infestation. The most admixed population is Bonn-Hoholz, followed by Linz and Altenrath, instead of those populations closest to Belgium (Walberberg) or Switzerland (Bad Hönningen). It therefore seems that genotype 1 specimens were transported from Belgium, Switzerland and/or Austria/Slovenia (or even possibly other locations where genotype 1 may have established) into Bonn, a medium sized city on the margins of the Rhine River in the German federal state of North Rhine-Westphalia, where they admixed with a local population of *Ae. j. japonicus*, which originally had a very distinct signature both in its *nad*4 composition and genotypic makeup (genotype 2). The population with a genotype 2 signature with the least admixed specimens and a low genetic diversity is Bad Hönningen, which of the five locations studied is the farthest from Bonn (Fig. 1). This indicates that introductions of both genotypes (first genotype 2 and subsequently genotype 1) may have occurred into the Bonn area.

Clearly our data show that western German *Ae. j. japonicus* are not simply an expansion of the Belgium population, as we first thought possible because of the geographical adjacency. Instead our results indicate that the population of *Ae. j. japonicus* that was first detected in Belgium in 2002 has not expanded on its own, possibly due to low genetic diversity (this population had the lowest genetic diversity of all populations examined, Fig. 3). However, the presence of genotype 1 specimens, with similar genotypic and haplotypic signatures in Belgium, Switzerland and Austria/Slovenia opens the possibility that all three introductions were derived from the same source of *Ae. j. japonicus.* At this point it is unclear if the Belgium populations were really “the first” or just “the first found”. In any case, following human-mediated transport from its source in northeast Asia to somewhere in Europe where it established, genotype 1 was moved to multiple locations in Europe. Alternatively, there could have been multiple introductions and establishments from the same source in Asia over the years. In either scenario the fact that the distribution of genotype 1 is very discontinuous indicates that the expansion of *Ae. j. japonicus* in Europe has been predominantly human-mediated.

Critically, we found undisputed evidence of a second genotype of *Ae. j. japonicus* in Europe, possibly introduced to or near Bonn in western Germany. And we found evidence that in areas where specimens from genotype 1 encountered specimens from genotype 2, admixed populations with increased genetic diversity were produced. These appear to be invasive, i.e. capable of spreading unaided, since the species now occurs continuously in a broad region in Germany from the state of North Rhine-Westphalia into Rhineland-Palatinate and potentially into Baden-Württemberg, although a detailed analysis of specimens from that state will be required to test this hypothesis. In conclusion, we found evidence of at least two different introductions of *Ae. j. japonicus* to Europe resulting in two unique genetic signatures and an expanding population with clear signs of admixture of the two (Figs. 2 and 3).

These analyses, of course, do not reveal the origin of European *Ae. j. japonicus*. They may have arrived from sources in Asia or from the USA where the species is now relatively abundant [11], although haplotype H5, commonly associated with the European genotype 2, is very rare in the US and H33, associated with genotype 1, has not been detected there [9], which indicates that Asia may be the more likely source of the European specimens. Assessing origin will require a thorough comparative analysis of representative populations of *Ae. j. japonicus* across the world.

Regarding the expansion across Europe, however, and especially considering the known distribution of genotype 1, we speculate that the Rhine River may have played an important role because it provides a traffic artery between industrial sites in the Netherlands in the north all the way across western Germany to the Swiss border (Fig. 1). Importantly, our results also indicate that for the last decade human-mediated transport has been the main driver of the expansion of *Ae. j. japonicus* across Europe. However, if the recent seemingly fast expansion across Germany is a true event and not just the result of increased surveillance, then the evident mixing between the two genotypes may have changed something important in the characteristics of the European populations. After a decade of relatively slow expansion, *Ae. j. japonicus* abundance and continuous occurrence from Zurich in Switzerland to Bonn in Germany and even potentially into Hanover in northern Germany [54] and the Netherlands where the species was just detected [55] indicate populations that are expanding unaided, which may complicate control measures considerably.

Although in Japan this mosquito is not considered an important nuisance or disease vector [14], US populations of *Ae. j. japonicus* have risen to nuisance levels especially in more northern states such as Michigan [11], which are too cold for urban vectors such as *Ae. albopictus* and *Culex pipiens.* Their willingness to bite humans is underscored by the fact that 30% of the blood meals identified from *Ae. j. japonicus* from New Jersey suburbs were human with predominance of blood meals from large vertebrates such as deer [56]. Their preference for large vertebrates is especially worrisome due to the proximity and extensive trade between Europe and Africa where Rift Valley fever, a disease of ruminants that can be deadly to humans, is endemic [57]. Because US populations of *Ae. j. japonicus* have been shown to be highly competent vectors of Rift Valley fever virus [26] the presence of large populations of *Ae. j. japonicus* in northern Europe increases the likelihood of Rift Valley fever epidemics, similar to the danger created by the presence of large populations of *Ae. albopictus* in southern Europe. Indeed, the abundance of *Ae. albopictus* in Italy has already resulted in local transmission of chikungunya virus [58]. “Human intervention” regarding *Ae. j. japonicus* needs to cease to be accidental and instead become deliberate and organized. Our results indicate that a first and critical step towards managing *Ae. j. japonicus* is increased surveillance and active control to identify and stop further introductions, establishments and mixing of differentiated populations.

## Supporting Information

Figure S1 **Results of a factorial correspondence analysis performed on individual genotypes in Genetix 4.05.** Yellow squares and burgundy squares correspond to individuals from Belgium and Austria/Slovenia populations, respectively. Swiss specimens are shown in dark blue, the remaining colors (light blue, pink, green, grey and white) are from German populations. These results mirror the results of the principal coordinate analysis on populations although it is hard to separate German populations, which is not surprising since they all have some degree of admixture between two introductions. Of note the green squares correspond to specimens from Bad Hönningen, which have the lowest genetic diversity (lowest levels of admixture).
(JPG)
