# Correction: Unexpected Patterns of Admixture in German Populations of *Aedes japonicus japonicus* (Diptera: Culicidae) Underscore the Importance of Human Intervention

**DOI:** 10.1371/journal.pone.0109471

**Published:** 2014-09-25

**Authors:** The PLOS ONE Staff

The word " japonicus" is misspelled in the article’s short title. The correct short title is: *Aedes japonicus* Population Genetics in Germany.

Additionally, the following paragraph was incorrectly placed as the last paragraph of the Materials and Methods section. It should appear as the last paragraph of the Results section:

The distribution of nad4 haplotypes matches to some extent the distribution of the two genotypes. Specifically, haplotype H5 occurs exclusively and is highly abundant in populations with a predominant genotype 2 signature (Fig. 1).
