# Supplementary figures and images for: Unexpected Patterns of Admixture in German Populations of Aedes japonicus japonicus (Diptera: Culicidae) Underscore the Importance of Human Intervention
Source: PLoS One. 2014 Jul 3;9(7):e99093. doi: 10.1371/journal.pone.0099093 (PMC4081119; doi:10.1371/journal.pone.0099093)

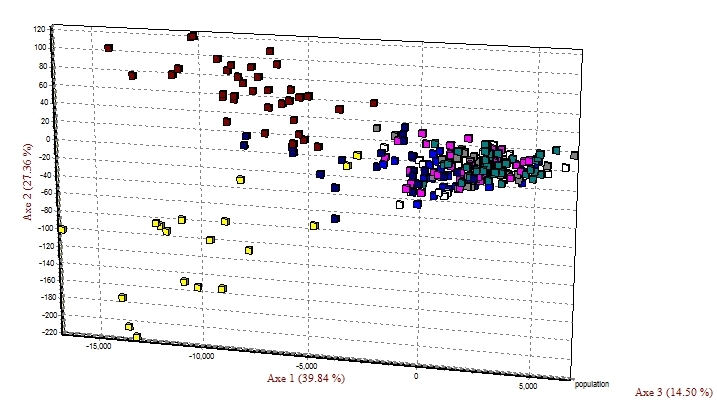

Supplement: Figure S1 — Results of a factorial correspondence analysis performed on individual genotypes in Genetix 4.05. Yellow squares and burgundy squares correspond to individuals from Belgium and Austria/Slovenia populations, respectively. Swiss specimens are shown in dark blue, the remaining colors (light blue, pink, green, grey and white) are from German populations. These results mirror the results of the principal coordinate analysis on populations although it is hard to separate German populations, which is not surprising since they all have some degree of admixture between two introductions. Of note the green squares correspond to specimens from Bad Hönningen, which have the lowest genetic diversity (lowest levels of admixture). (JPG) [file pone.0099093.s001.jpg]
